# Seroprevalence of Foot and Mouth Disease Virus Infection in Some Wildlife and Cattle in Bauchi State, Nigeria

**DOI:** 10.1155/2020/3642793

**Published:** 2020-03-18

**Authors:** Y. J. Atuman, C. A. Kudi, P. A. Abdu, O. O. Okubanjo, A. Abubakar, Y. Wungak, H. G. Ularamu

**Affiliations:** ^1^National Veterinary Research Institute Vom Outstation Laboratory, Bauchi, Bauchi, Nigeria; ^2^Department of Veterinary Medicine, Faculty of Veterinary Medicine, Ahmadu Bello University, Zaria, Nigeria; ^3^Department of Veterinary Parasitology and Entomology, Ahmadu Bello University, Zaria, Nigeria; ^4^Force Animal Branch Department, Nigeria Police Force Headquarters, Abuja, Nigeria; ^5^Viral Research Division, National Veterinary Research Institute, Vom, Nigeria

## Abstract

Foot and mouth disease (FMD) is an important transboundary viral disease of both domestic and wild cloven-hoofed animals characterized by high morbidity with devastating consequence on the livestock worldwide. Despite the endemic nature of FMD in Nigeria, little is known about the epidemiology of the disease at the wildlife-livestock interface level. To address this gap, blood samples were collected between 2013 and 2015 from some wildlife and cattle, respectively, within and around the Yankari Game Reserve and Sumu Wildlife Park in Bauchi State, Nigeria. Wild animals were immobilized using a combination of etorphine hydrochloride (M99® Krüger-Med South Africa) at 0.5–2 mg/kg and azaperone (Stresnil®, Janssen Pharmaceuticals (Pty.) Ltd., South Africa) at 0.1 mg/kg using a Dan-Inject® rifle (Dan-Inject APS, Sellerup Skovvej, Denmark) fitted with a 3 ml dart syringe and for reversal, naltrexone (Trexonil® Kruger-Med South Africa) at 1.5 mg IM was used, and cattle were restrained by the owners for blood collection. Harvested sera from blood were screened for presence of antibodies against the foot and mouth disease virus (FMDV) using the PrioCHECK® 3ABC NSP ELISA kit, and positive samples were serotyped using solid-phase competitive ELISA, (IZSLER Brescia, Italy). Out of the 353 sera collected from cattle and wildlife 197 (65.7%) and 13 (24.5%) (*P* < 0.05), respectively, tested positive for antibodies to the highly conserved nonstructural 3ABC protein of FMDV by the FMDV-NS blocking ELISA. Classification of cattle into breed and sex showed that detectable antibodies to FMDV were higher (*P* < 0.05) in White Fulani 157 (72.8%) than in Red Bororo 23 (39.7%) and Sokoto Gudali 17 (33.3%) breeds of cattle, whereas in females, detectable FMDV antibodies were higher (*P* < 0.05) 150 (72.8%) than in males 47 (50.0%). In the wildlife species, antibodies to FMDV were detected in the waterbucks 2 (28.6%), elephant 1 (25.0%), wildebeests 4 (33.3%), and elands 6 (25.0%). Four serotypes of FMDV: O, A, SAT 1, and SAT 2 were detected from the 3ABC positive reactors in waterbucks, elephants, wildebeests, and elands. The results showed presence of antibodies to FMDV in some wildlife and cattle and suggested that wildlife could equally play an important role in the overall epidemiology of FMD in Nigeria. FMD surveillance system, control, and prevention program should be intensified in the study area.

## 1. Introduction

Foot and mouth disease (FMD) is one of the most economically important transboundary animal disease in the world caused by foot and mouth disease virus (FMDV), a member of the genus *Aphthovirus* belonging to the *Picornaviridae* family [1]. FMDV is a small nonenveloped virus and has a genome of 8.5 kb which encodes for structural proteins (VP1, VP2, VP3, and VP4) as well as nonstructural proteins (NSPs) [2, 3]. A structural protein produces antibodies to FMDV in vaccinated animals, whereas infected animals produce antibodies to both the structural and nonstructural proteins [3], and assays to demonstrate antibodies against nonstructural proteins have potential to differentiate infected from vaccinated animals [4–7]. Seven immunologically different serotypes of the FMDV are known: O, A, C, Asia-1, and South-African Territories (SAT) 1, 2, and 3, which comprise more than 65 subtypes [8].

The transmission of FMDV in sub-Saharan Africa is mainly driven by two epidemiological cycles: one in which wildlife plays a significant role in maintaining and spreading the disease to other susceptible wild and/or domestic ruminants [9, 10]. Whilst with the second cycle, the virus is solely transmitted within domestic populations and hence is independent of wildlife [11]. FMD is currently found in limited areas (small pockets/regions) of Europe and also in Africa, Middle East, and Asia and has contributed to significant declines in wildlife and livestock populations in those regions [12–15]. The first reported case of FMD outbreak in Nigeria was in 1924, which was attributed to type O virus [16]. Subsequently, other serotypes (A, SAT 1, and SAT 2) were reported [17–22] and recently SAT 3 serotype [23].

In spite of the annual FMD burden in Nigeria, seroepidemiology and serotyping studies for FMD infections are inadequate. The current trend of FMD occurrence in Nigeria showed that there are regular outbreaks, poor control measures, and lack of enforcement of legislation guiding disease reporting to veterinary authority [24, 25]. The presence of antibodies to FMDV in several wildlife species has been documented in studies conducted in different countries of Africa mainly eastern and southern regions [26–28]. There has been limited monitoring of infectious diseases like FMD in wildlife in Nigeria. Domestic livestock sometimes do share the same range with wildlife in YGR and SWP in Bauchi State, Nigeria [29], and there is concern that wildlife may form a reservoir for FMDV. Consequently, there is a need to understand the potential role of wildlife as reservoir of FMDV to aid in the design and implementation of the disease management programs. The aim of the study was to determine the seroprevalence of FMDV in wildlife and cattle and identify circulating FMDV serotypes in wildlife in YGR and SWP in Bauchi State, Nigeria.

## 2. Materials and Methods

### 2.1. Study Area

**Figure 1:**
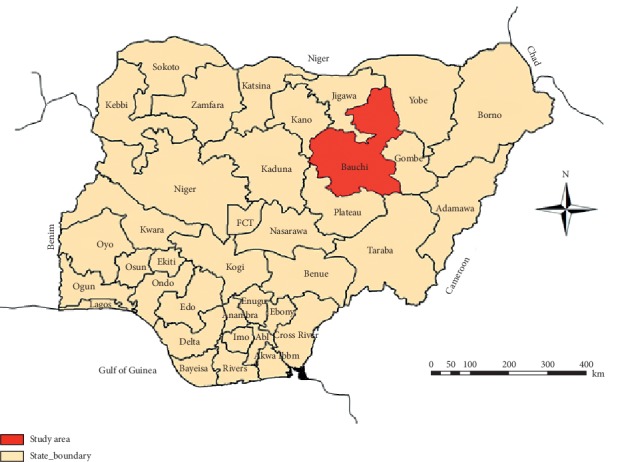
Map of Nigeria showing Bauchi State. Source: Modified from the Administrative Map of Nigeria (http://www.theodora.com/maps).

**Figure 2:**
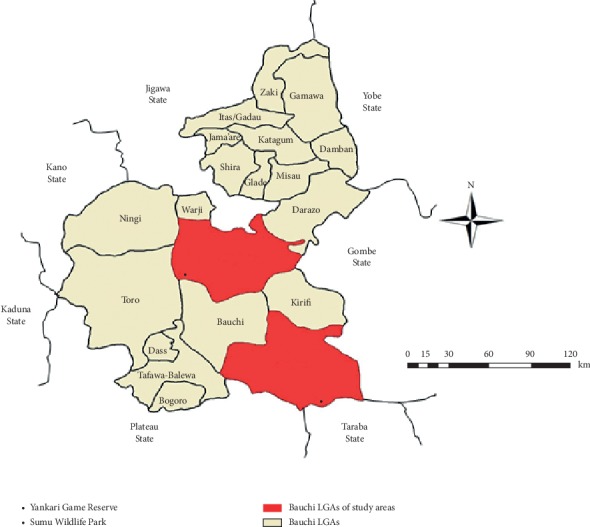
Map of Bauchi State showing study areas. Source: Modified from the Administrative Map of Nigeria (http://www.theodora.com/maps).

The study locations were YGR and SWP in Bauchi State, Nigeria (Figures 1 and 2). The YGR covers an area of about 2,244 square kilometers and it is an important refuge for over 50 species of mammals and over 350 species of birds and is one of the few remaining areas where wild animals are protected in their natural habitat in Nigeria [30, 31], whereas SWP covers about 40 square kilometer area and harbours species of wildlife including impala (*Aepyceros melampus*), springbok (*Antidorcas marsupialis*), oryx (*Oryx gazelle*), common eland (*Taurotragus oryx*), zebra *(Equus quagga crawshayi*), greater kudu (*Tragelaphus strepsiceros*), blue wildebeest (*Connochaetes taurinus*), and giraffe (*Giraffa camelopardalis*) and is located about 60 km north of the state capital, Bauchi [29].

### 2.2. Sampled Animals

Wildlife samples included elephant (*Loxodonta africana*), waterbuck (*Kobus ellipsiprymus*), and hartebeest (*Alcelaphus buselaphus caama*) from YGR and eland (*Taurotragus oryx*), kudu (*Tragelaphus strepsiceros*), and blue wildebeest (*Connochaetes taurinus*) from SWP. Cattle were sampled from herds of cattle located at the fringes of the YGR and SWP.

### 2.3. Sample Collection

Wildlife were immobilized for sample collection using a combination of etorphine hydrochloride (M99® Krüger-Med South Africa) at 0.5–2 mg/kg and azaperone (Stresnil®, Janssen Pharmaceuticals (Pty.) Ltd., South Africa) at 0.1 mg/kg delivered intramuscularly (IM) from a distance of about 25 meters on ground in a 3 ml dart syringe fitted with barbed needles using a Dan-Inject® rifle (Dan-Inject APS, Sellerup Skovvej, Denmark). Cattle were restrained by the owners for sample collection. Ten mililitres of blood samples were collected from the jugular vein of each animal and dispensed into plain vacutainer bottles. All samples were transported in a cold box with ice packs to the National Veterinary Research Institute Laboratory, Bauchi. The serum samples were harvested from the blood into cryovials after spinning for 10 min at 1200 g and were divided into aliquots, labelled, and kept at −20°C until used.

### 2.4. Detection of Antibodies against FMDV Nonstructural Proteins (NSPs) by ELISA

The ELISA was performed according to the manufacturer's instructions (PRIOCHECK® FMD-3ABC NS protein ELISA) for detection of antibodies to the nonstructural polypeptide 3ABC of FMDV in serum which detects infected animals regardless of their vaccination status and the FMDV serotype that caused the infection [32]. Briefly, 80 *μ*l of the ELISA buffer and 20 *μ*l of the test sera were added to the 3ABC antigen-coated test plates. Negative, weak positive, and strong positive control sera were added to designated wells on each test plate, gently shaken, and incubated overnight (18 h) at 22°C. The plates were then emptied and washed six times with 200 *μ*l of wash solution, and 100 *μ*l of diluted conjugate was added to all wells. The test plates were sealed and incubated for one hour at 22°C. The plates were then washed six times with 200 *μ*l of wash solution, and 100 *μ*l of the chromogen (tetramethylbenzidine) substrate was dispensed to all wells of the plates and incubated for 20 min at 22°C following which 100 *μ*l of the stop solution was added to all the wells and mixed gently. Readings were taken on a spectrophotometer Multiskan® ELISA reader (Thermo Scientific, USA) at 450 nm, and the OD 450 values of all samples were expressed as percentage inhibition (PI) relative to the OD 450 max using the following formula PI = 100 − [OD 450 test sample/OD450 max] × 100. Samples with PI = ≥50% were considered positive for the FMD antibody, while those with PI <50% were declared negative for the FMD antibody. Since the 3ABC ELISA for FMD was = 100% specific and >99% sensitive, the percentage prevalence was taken as true prevalence.

### 2.5. Detection of FMDV-Specific Antibodies Using Solid-Phase Competitive Enzyme-Linked Immunosorbent Assay

The 3ABC ELISA positive serum samples were analyzed for FMD-specific antibodies using a solid-phase competitive ELISA (SPCE) as previously described for serotypes O, A, SAT 1, and SAT 2 [32, 33]. The assays were performed using antibodies FMDV ELISA kits for serotypes O, A, SAT 1, and SAT 2 produced by IZSLER Biotechnology Laboratory (Italy). Briefly, 96 wells precoated with FMDV antigens captured by FMD serotypes O, A, SAT 1, and SAT 2 in specific MAb flat-bottomed plates were used. Four dilutions of sera at 1/10, 1/30, 1/90, and 1/270 were made. Without washing, the conjugate (horse-radish peroxidase) was added and incubated at room temperature for 1 h. The plate was washed, and the substrate/chromogen solution (tetramethylbenzidine) was added and kept in the dark for 20 min. The reaction was stopped by the addition of a stop solution, and the plates were read on a MultiSkan® spectrophotometer ELISA plate reader (Thermo Scientific, USA) at 450 nm wavelength. Serum endpoint titre was expressed as the highest dilution producing 50% inhibition, with serum having endpoint titre ≥50% being classified as positive for the specific FMD antibody.

Data obtained were analyzed using Graphpad Prism version 7. Results were summarized in tables and expressed as percentages and levels of association between positivity and sex, breed, and age, and animal species were derived using chi-square. Values of *P* ≤ 0.05were regarded as statistically significantly different.

## 3. Results

Overall seroprevalence of FMDV in wildlife was 24.5% (Table 1). Detectable antibodies to FMDV were observed in the waterbuck 28.6%, elephant 25.00 %, wildebeest 33.3%, and eland 25.0 %

Comparison of the overall seroprevalences of FMDV at the wildlife-cattle interface (Table 2) showed that detectable antibodies to FMDV were significantly higher (*P* < 0.05) in cattle 65.67% than in wildlife 24.0%.

Antibodies to FMDV were significantly higher in female cattle than males (*P* <0.05) with Bunaji breed of cattle having a high risk factor (odds ratio >5) of exposure to FMDV than the other breeds of cattle examined (Table 3).

The detectable antibodies to the FMD serotype were for serotypes O, A, SAT 1, and SAT 2 in waterbuck, wildebeest, and eland, whereas antibodies to serotypes A and SAT 2 were detected in elephants. Each of the serotypes A and SAT 1 was shown to have highest reactors of 18.87%, whereas serotype O had the least reactor of 13.21% (Table 4).

## 4. Discussion

The results of this study have shown that antibodies to FMDV were present in cattle (65.7%) and wildlife (24.5%). This is consistent with results of previous survey for FMDV antibodies in Nigeria in which a seroprevalence of 75.11% was reported in a study conducted in cattle in Kwara State [34]. Also, seroprevalences of 64.3% and 70.98%, respectively, were reported in studies carried out in Plateau State [35, 36] and 64.7% in a study conducted at the border states in Nigeria [21, 37]. The similarities of findings of the present study with previous studies have shown that FMD is still an enzootic disease in Nigeria, and this could be attributed to the lack of FMD vaccination campaigns in Nigeria [21, 37]. There is unrestricted herds mobility, continuous contact, and intermingling of different cattle herds at water points, communal grazing areas, and porous borders.

The higher FMDV seroprevalence in female cattle during this study was consistent with the findings of other investigators [34, 37] which reported a risk difference in association with sex during FMDV studies in Kwara and Plateau states, Nigeria, respectively. Similarly, high incidence of FMDV in females in Northwest Ethiopia was reported [38]. However, most of the cattle sampled during the study were females as opposed to males. The significant association of seroprevalence with sex could be attributed to the preference for females to males by the nomads for reproductive purposes and milk production, and therefore females are kept for a longer period thereby having higher risk of exposure than males [8, 34, 37]. Significant association in seropositivity was observed in the Bunaji breed of cattle, and this could be due to small number of other breeds (Sokoto Gudali and Red Bororo) sampled. However, all the breeds of cattle are equally at risk.

Results from the study have shown that antibodies to FMDV were present in elands, wildebeests, waterbucks, and elephants. This finding being the first of its kind in the study area reveals that FMD could be a problem in wildlife in Nigeria. This is not surprising as FMD is endemic in Nigeria [18, 20, 23, 39]. Presence of wildlife population along the national park in Borgu, Niger State, Nigeria, where cloven-hoofed species come in contact with livestock was shown to be the probable exposure factor that contributed to high FMD seropositivity in livestock observed in the area [37]. The results from this study corroborate with other studies in South Africa, Zimbabwe, Zambia, Botswana, Namibia, India, Chad, and Iran that demonstrated FMDV antibodies in wildlife [10, 11, 28, 40–45]. High FMDV prevalence in waterbucks observed in this study reflects their ecology and living ecosystem which is consistent with other findings in East Africa and Zimbabwe [27, 41, 46]. The study hitherto provided a picture of FMDV distribution in wildlife in Bauchi State, Nigeria, which was observed to be largely understudied [44].

This study confirms the presence of an antibody to FMDV serotypes O, A, SAT 1, and SAT 2 in wildlife, a finding which is first of its kind in Bauchi State, Nigeria. Reported outbreaks affecting livestock of West Africa since 2000 were caused by FMDV types O, A, and SAT 2 [44]. Similarly, FMDV serotypes O, A, and SAT 2 were the cause of most reported outbreaks in domestic livestock in Nigeria from 2010 to 2016 [22, 34, 39]. The result here showed that FMDV serotypes observed in wildlife were equally previously observed in domestic livestock. The possible source of FMDV serotypes infection for the wildlife could be from infected livestock interacting with wildlife in the same environment. Transmission of FMDV between wildlife and livestock, even in isolated areas, may be due to windborne infection or via fomites [47, 48]. Wildlife species often congregate at the natural “salt lick” point in YGR [31]; similarly, artificial salt lick points are also available in SWP. Therefore, dissemination of the FMDV during wildlife activities at the salt lick points is possible. Previous studies have shown that FMDV can easily be disseminated in the soil and can persist in that environment for a long period [28].

The presence of FMDV antibodies in wildlife and cattle in this study might be driven by direct contact at the wildlife-livestock interface through sharing of water and pasture resources which is observed to be a common activity in YGR and SWP in Bauchi State, Nigeria [29, 31]. During dry season, wildlife and livestock in the study area do closely congregate at feed and water points thus increasing the transmission likelihood of water-related infections like FMD [13, 41, 44]. Studies conducted in Ethiopia and Zimbabwe found significant association between cattle exposed to FMDV and their contact history with wildlife [11, 48, 49]. It is unfortunate that due to the endemic nature of FMD in Nigeria that outbreaks are not being investigated to determine the primary source, and hence the disease has continued to be a scourge to livestock production in the country.

## 5. Conclusion

Presence of FMDV antibodies in both cattle and some wildlife was observed. Also, four serotypes of FMDV: O, A, SAT 1, and SAT 2 previously detected in cattle in Nigeria were observed for the first time among some wildlife species in the study area. The study highlights the implication of continuous spread of FMD due to access to resources like water and grazing areas shared by wildlife and livestock in the study area. This is a threat to livestock production and wildlife conservation goals, and hence there is a need for adapting livestock and or wildlife management practices that will reduce the frequency of disease transmission at the wildlife and livestock contact. Further studies are needed to isolate and characterize the FMD-circulating virus in wild and domestic animals from the study area.

## Figures and Tables

**Table 1:** Seroprevalence of the foot and mouth disease virus in wildlife from Yankari Game Reserve and Sumu Wildlife Park in Bauchi State, Nigeria.

Wildlife	No. sampled (%)	No. +ve (%)	*X* ^2^	*P* value	Odds ratio	CI at 95%
Yankari Park Waterbuck	11 (10.4)	2 (18.2)	1.395	0.943	0.373	0.899–0.327
Elephant	4 (3.8)	1 (25.0)				
Hartebeest	1(0.9)	0 (0.0)				
Sumu Park Eland	24 (22.6)	6 (25.0)				
Wildebeest	12 (11.3)	4 (33.3)				
Kudu	1 (0.9)	0 (0.0)				
Overall	53 (100)	13 (24.5)				

**Table 2:** Seroprevalence of foot and mouth disease at the wildlife-cattle interface in Yankari Game Reserve and Sumu Wildlife Park in Bauchi State, Nigeria.

Species	No. sampled	No. +ve (%)	*X* ^2^	*P* value	Odds ratio	CI at 95%
Wildlife	53	13 (24.53)	31.63	0.000	0.1699	0.087–0.332
Cattle	300	197 (65.67)				
Overall	353	210 (59.49)				

**Table 3:** Seroprevalence of the foot and mouth disease virus in cattle around Yankari Game Reserve and Sumu Wildlife Park in Bauchi State, Nigeria.

Variables	No. sampled (%)	No +ve (%)	*X* ^2^	*P* value	Odds ratio	CI at 95%
*Breed*						
Red Bororo	58 (19.3)	23 (39.7)	64.2	0.000		
Sokoto Gudali	51 (17.0)	17 (33.3)			0.544	0.241–1.225
White Fulani	191 (63.7)	157 (82.2)			5.019	2.550–9.878
Overall	300 (100)	197 (65.7)				
*Sex*						
Male	94 (31.3)	47 (50.0)	14.9	0.000	0.373	0.225–0.620
Female	206 (68.7)	150 (72.8)				
Overall	300 (100.0)	197 (65.7)				

**Table 4:** Foot and mouth disease virus serotypes detected in wildlife in Yankari Game Reserve and Sumu Wildlife Park in Bauchi State, Nigeria.

Wildlife	No. tested (%)	Foot and mouth disease virus serotypes			
		O	A	SAT 1	SAT 2
		No. positive (%)			
Waterbuck	11 (10.4)	2 (18.18)	2 (18.18)	3 (27.27)	1 (9.09)
Elephant	4 (3.8)	0	1 (25.0)	0	1 (25.0)
Hartebeest	1 (0.9)	0	0	0	0
Eland	24 (22.6)	3 (12.5)	6 (25.0)	4 (16.67)	4 (16.67)
Wildebeest	12 (11.3)	2 (16.67)	1 (8.33)	3 (25.0)	2 (16.67)
Kudu	1 (0.9)	0	0	0	0
Overall	53 (100)	7 (13.21)	10 (18.87)	10 (18.87)	8 (15.09)

## Data Availability

All relevant data are included in the tables, and should there be need of any additional data, they will be provided accordingly.

## References

[1] Brown C. C., Slenning B. D. (1996). Impact and risk of foreign animal diseases. *Journal of the American Veterinary Medical Association*.

[2] Fry E. E., Stuart D. I., Rowlands D. J. (2005). The structure of foot-and-mouth disease virus. *Current Topics in Microbiology and Immunology*.

[3] Yousef M., Mazloum K., AlNakhli H. (2012). Serological evidence of natural exposure of camels camelus dromedaries to foot and mouth disease virus. *Veterinary World*.

[4] Berger H.-G., Straub O. C., Ahl R., Tesar M., Marquardt O. (1990). Identification of foot-and-mouth disease virus replication in vaccinated cattle by antibodies to non-structural virus proteins. *Vaccine*.

[5] Rodriguez A., Dopazo J., Saiz J. C., Sobrino F. (1994). Immunogenicity of non-structural protein of foot-and-mouth disease virus; differences between infected and vaccinated swine. *Archive of Virology*.

[6] De Diego M., Brocchi E., Mackay D., De Simone F. (1997). THE non-structural polyprotein 3ABC of foot-and-mouth disease virus as a diagnostic antigen in ELISA to differentiate infected from vaccinated cattle. *Archives of Virology*.

[7] Clavijo A., Wright P., Kitching P. (2004). Developments in diagnostic techniques for differentiating infection from vaccination in foot-and-mouth disease. *The Veterinary Journal*.

[8] Longjam N., Deb R., Sarmah A., Tayo T., Awachat V., Saxena V. (2011). A brief review on diagnosis of foot-and-mouth disease of livestock: conventional to molecular tools. *Veterinary Medicine International*.

[9] Dawe P., Flanagan F., Madekurozwa R. (1994). Natural transmission of foot-and-mouth disease virus from African buffalo (*Syncerus caffer*) to cattle in a wildlife area of Zimbabwe. *Veterinary Record*.

[10] Bastos A. D. S., Boshoff C. I., Keet D. F., Bengis R. G., Thomson G. R. (2000). Natural transmission of foot-and-mouth disease virus between African buffalo (Syncerus caffer) and impala (*Aepyceros melampus*) in the Kruger National Park, South Africa. *Epidemiology and Infection*.

[11] Miguel E., Grosbois V., Caron A. (2013). Contacts and foot and mouth disease transmission from wild to domestic bovines in Africa. *Ecosphere*.

[12] Daszak P., Cunningham A. A., Hyatt A. D. (2000). Emerging infectious diseases of wildlife–threats to biodiversity and human health. *Science*.

[13] Bengis R. G., Leighton F. A., Fischer J. R., Artois M., Morner T., Tate C. M. (2004). The role of wildlife in emerging and re-emerging zoonoses. *Revue Scientifique et Technique-Office International des Epizooties*.

[14] Pedersen A. B., Jones K. E., Nunn C. L., Altizer S. (2007). Infectious diseases and extinction risk in wild mammals. *Conservation Biology*.

[15] Smith M. J., Telfer S., Kallio E. R. (2009). Host-pathogen time series data in wildlife support a transmission function between density and frequency dependence. *Proceedings of the National Academy of Sciences*.

[16] Libeau J. (1960). Foot-and-mouth disease in Africa south of the Sahara the present situation. *Bulletin of Epizootic Diseases in Africa*.

[17] Nawathe D. R., Goni M. (1976). Foot and mouth disease in Nigeria. *Bulletin of Animal Health and Production in Africa*.

[18] Owolodun B. (1971). Foot-and-mouth disease and virus types distribution in Nigeria. *Bulletin of Epizootic Diseases of Africa*.

[19] Durojaiye A. (1981). Incidence of FMD in oyo state of Nigeria, 1967–1981. *Nigeria Veterinary Journal*.

[20] Abegunde A. A., Ezeokoli C. D., Umoh J. U., Addo P. B., Williams O., Masiga W. N. (1988). Relationship between recent FMD virus isolates from Nigeria and standard vaccine virus strain from the African region. *Viral Diseases of Animals in Africa*.

[21] Lazarus D. D., Schielen W. J. G., Wungak Y. S., Kwange D. Y., Fasina F. O. (2012). Sero-epidemiology of foot-and-mouth disease in some Border States of Nigeria. *African Journal of Microbiology Research*.

[22] Ularamu H. G., Ibu J. O., Wood B. A. (2017). Characterization of foot-and-mouth disease viruses collected in Nigeria between 2007 and 2014: evidence for epidemiological links between West and East Africa. *Transboundary and Emerging Diseases*.

[23] Ehizibolo D. O., Haegeman A., De Vleeschauwer A. R. (2017). Detection and molecular characterization of foot and mouth disease viruses from outbreaks in some states of northern Nigeria 2013–2015. *Transboundary and Emerging Diseases*.

[24] Lazarus D. D., Wungak Y. S., Adah M. I. (2015). Serological response of commercial dairy cattle to inactivated foot-and-mouth disease vaccine (type-O & A) in Nigeria. *Asian Journal of Medical and Biological Research*.

[25] Vandenbussche F., Mathijs E., Ularamu H. G. (2018). Complete genome sequences of five foot-and-mouth disease viruses of serotype a isolated from cattle in Nigeria between 2013 and 2015. *Genome Announcements*.

[26] Williams E. S., Yuill T., Artois M., Fischer J., Haigh S. A. (2002). Emerging infectious diseases in wildlife. *Revue Scientifique et Technique de l’OIE*.

[27] Bronsvoort B. M. D. C., Parida S., Handel I. (2008). Serological survey for foot-and-mouth disease virus in wildlife in eastern Africa and estimation of test parameters of a nonstructural protein enzyme-linked immunosorbent assay for buffalo. *Clinical and Vaccine Immunology*.

[28] Vosloo W., Thompson P. N., Botha B., Bengis R. G., Thomson G. R. (2009). Longitudinal study to investigate the role of impala (*Aepyceros melampus*) in foot-and-mouth disease maintenance in the Kruger national park, South Africa. *Transboundary and Emerging Diseases*.

[29] Atuman Y., Adawa Y., Solomon A., Mshelbwala P., Ogunkoya A. (2014). Potential risks for rabies spill-over from apparently healthy dogs to wildlife in Bauchi state, Nigeria. *Journal of Veterinary Advances*.

[30] Odunlami S. S., Lake B. (2003). An assessment of the ecotourism potential of Yankari national park, Nigeria. *Eco Club. Communication E paper Series Nr. 7*.

[31] Omondi P., Mayienda R., Mshelbwala J. H., Massalatchi M. S. (2006). Total aerial count of elephants, buffaloes, roan antelope and other wildlife species in Yankari ecosystem, Nigeria.

[32] Ularamu H. G., Ibu J. O., Abenga J. N. (2017). Improving laboratory capacity for foot and mouth disease diagnosis and control for sustainable livestock production in Nigeria. *Vom Journal of Veterinary Science*.

[33] Chénard G., Miedema K., Moonen P., Schrijver R. S., Dekker A. (2003). A solid-phase blocking ELISA for detection of type O foot-and-mouth disease virus antibodies suitable for mass serology. *Journal of Virological Methods*.

[34] Olabode O., Kazeem H., Raji M., Ibrahim N. (2013). Seroprevalence of foot and mouth disease virus antibodies in trade cattle (*Bos indicus*) in Kwara state of Nigeria. *Veterinary World*.

[35] Ehizibolo D. O., Ajogi I., Umoh J. U. Serological survey of foot and mouth disease (FMD) 3D non-structural proteins using virus-infection associated (VIA) antigen assay in livestock animals from Plateau State, Nigeria.

[36] Wungak Y. S., Olabisi I. O., Olugasa B. O., Lazarus D. D., Ularamu H. G. (2015). Seroprevalence of foot and mouth disease (FMD) among sedentary cattle in northern Plateau, Nigeria. *Asian Journal of Medical and Biological Research*.

[37] Wungak Y. S., Olugasa B. O., Ishola O. O., Lazarus D. D., Ularamu G. H. (2016). Foot-and-mouth disease (FMD) prevalence and exposure factors associated with seropositivity of cattle in north-central, Nigeria. *African Journal of Biotechnology*.

[38] Mazengia H., Taye M., Negussie H., Alemu S., Tassew A. (2010). Incidence of foot and mouth disease and its effect on milk yield in dairy cattle at Andassa dairy farm, Northwest Ethiopia. *Agriculture and Biology Journal of North America*.

[39] Chukwuedo A., Nimzing L. (2012). Field investigation of Foot and Mouth Disease (FMD) virus infection in cattle in the northern states of Nigeria. *Nigerian Journal of Biotechnology*.

[40] Esterhuysen J. J., Thomson G. R., Flammand J. R., Bengis R. G. (1985). Buffalo in the Natal game parks show no serological evidence of infection with Foot and mouth disease virus. *Ondersteeport Journal of Veterinary Research*.

[41] Sutmoller P., Thomson G. R., Hargreaves S. K., Foggin C. M., Anderson E. C. (2000). The foot-and-mouth disease risk posed by African buffalo within wildlife conservancies to the cattle industry of Zimbabwe. *Preventive Veterinary Medicine*.

[42] Thomson G. R., Vosloo W., Bastos A. D. S. (2003). Foot and mouth disease in wildlife. *Virus Research*.

[43] Vosloo W., De Klerk L.-M., Boshoff C. I. (2007). Characterisation of a SAT-1 outbreak of foot-and-mouth disease in captive African buffalo (*Syncerus caffer*): clinical symptoms, genetic characterisation and phylogenetic comparison of outbreak isolates. *Veterinary Microbiology*.

[44] Di Nardo A., Libeau G., Chardonnet B. (2015). Serological profile of foot-and-mouth disease in wildlife populations of West and Central Africa with special reference to Syncerus caffer subspecies. *Veterinary Research*.

[45] Hemmatzadeh F., Boardman W., Alinejad A., Hematzade A., Moghadam M. K. (2016). Molecular and serological survey of selected viruses in free-ranging wild ruminants in Iran. *PLoS One*.

[46] Weaver G. V., Domenech J., Thiermann A. R., Karesh W. B. (2013). Foot and mouth disease: a look from the wild side. *Journal of Wildlife Diseases*.

[47] Alexandersen S., Zhang Z., Donaldson A. I. (2002). Aspects of the persistence of foot-and-mouth disease virus in animals-the carrier problem. *Microbes and Infection*.

[48] Bolortsetseg S., Enkhtuvshin S., Nyamsuren D. (2012). Serosurveillance for foot-and-mouth disease in Mongolian gazelles (*Procapra gutturosa*) and livestock on the Eastern Steppe of Mongolia. *Journal of Wildlife Diseases*.

[49] Molla B., Ayelet G., Asfaw Y., Jibril Y., Ganga G., Gelaye E. (2010). Epidemiological study on foot-and-mouth disease in cattle: seroprevalence and risk factor assessment in South Omo zone, South-Western Ethiopia. *Transboundary and Emerging Diseases*.

